# Salivary cortisol and eye temperature changes during endurance competitions

**DOI:** 10.1186/s12917-021-02985-9

**Published:** 2021-10-14

**Authors:** Monica C. de Mira, Elsa Lamy, Rute Santos, Jane Williams, Mafalda Vaz Pinto, Pedro S. Martins, Patrícia Rodrigues, David Marlin

**Affiliations:** 1 MED – Mediterranean Institute for Agriculture, Environment and Development, Institute for Advanced Studies and Research, Universidade de Évora, Pólo da Mitra, Ap. 94, 7006-554 Évora, Portugal; 2 VALORIZA – Research Centre for Endogenous Resource Valorization, Edifício BioBIP, Campus Politécnico, 10, 7300-555 Portalegre, Portugal; 3 Hartpury University, Gloucester, GL19 3BE UK; 4 Departamento de Medicina Veterinária, Universidade de Évora, Núcleo da Mitra, Apartado, 94 7006-554 Évora, Portugal; 5 Departamento de Medicina Veterinária Portugal, Escola Universitária Vasco da Gama, Av. José R. Sousa Fernandes, Campus Universitário – Bloco B, Lordemão, 3020-210 Coimbra, Portugal; 6 David Marlin Consulting, AnimalWeb Ltd, Cambridge, CB4 0WZ UK

**Keywords:** Endurance riding, Eye temperature, Infrared thermography, Salivary cortisol, Performance, Equine

## Abstract

**Background:**

The purpose of this study was to investigate the usefulness of salivary cortisol (SC) and eye temperature measured by infrared thermography (IRT^ET^) as biomarkers to manage competitions more effectively and monitor horse welfare in endurance competitions. Based on previous studies, it was hypothesised that pre-exercise baseline SC and IRT^ET^ would be higher in younger or less experienced horses, and that post-exercise variation from baseline would be higher in the top finishers.

**Results:**

Salivary cortisol measured in 61 competing at qualifier 40 km and 80 km rides showed an abrupt variation (93–256% rise) of the baseline SC levels [median ± interquartile range (IQR) = 0.27 ng/dl ± 0.36] obtained at the Pre-Inspection (PI) into Vet Gate (VG)1 independently of the covered distance, but modest or even lower in the subsequent Vet Gates, e.g. VG2 or VG3. The IRT^ET^ measured concomitantly in 16 horses showed significant (*p* < 0.05) higher levels at the PI in less experienced horses participating in the 40 km ride (median ± IQR = 35.7 °C ± 1.4) than their counterparts in the 80 km ride (median ± IQR = 35.0 °C ± 1.5), but not SC. Baseline SC levels at the PI of horses classifying in the Top5 in the 40 km ride category were significantly (*p* < 0.05) higher median ± IQR = 0.90 ng/ml ±0.61) when compared to horses positioned from 10th position on (median ± IQR = 0.16 ng/ml ±0.40). A lower IRT^ET^ in the PI was correlated with better placement (*p* < 0.05) and those in the Top5 (median ± IQR = 33.9 °C ± 0.0) had a significantly (*p* < 0.5) higher variation (+ 10.65%) into the last VG.

**Conclusion:**

Pre-exercise baseline IRT^ET^ levels, but not SC, were higher in less experienced horses in the 40 compared to their counterparts in the 80 km ride competitions. SC and IRT^ET^ showed different indications according to the competition. In the40 km ride competition, higher baseline pre-exercise SC levels seemed to be linked to a better classification outcome. In contrast, in the 80 km ride horses, the higher IRT^ET^ variation from pre-exercise into final Vet Gate was the parameter associated with a better performance. A more controlled environment and a larger sample are needed to confirm these results and monitor horse welfare in competitions.

## Background

Endurance ride competitions are long-distance races of 40 to 160 km against the clock in phases that consist of a minimum of 16 to a maximum of 40 km, followed by a required rest period, at least equal in minutes to the distance in km of the competition [1]. Mandatory veterinary inspections, before (pre-inspection) and after each phase are performed is an assigned area called the Vet Gate (VG) to determine if competing horses are fit to compete or need to be eliminated to protect their integrity [2]. Despite the highest elimination rates among all equestrian sports [3–5] and the introduction of stricter rules by the Fédération Equestre Internationale (FEI) severe injuries still occur. This is not only unacceptable for today’s societal welfare standards towards equine athletes [6, 7] but also frustrating for veterinarians, who are often confronted by competitors with the subjectivity of a decision to eliminate a horse [8]. For these reasons and because horses, unlike humans, cannot vocalise distress or pain or make decisions for themselves, the possibility of utilising non-invasive and objective methods, such as gait sensors [9] and biomarkers would be instrumental to evidence-based management of the equine athlete’s welfare while competing. Cortisol determination using saliva and eye temperature measured by infrared thermography are non-invasive techniques used to evaluate horses’ stress responses to its human equestrian utilisation [10].

Exercise is a naturally a stressor per se and induces a biologic response that can be either an enhancer or a limiting factor for an athlete’s sporting ability [11]. Yet, in competitions, equine athletes face a mixture of other stressors including transportation [12], veterinary examinations [13], rider’s ability [14], a new and a noisy environment [15], separation from stablemates [16] and, specifically in endurance, exposure to large conglomerations of unfamiliar horses in large starts, and musculoskeletal pain from an injury that might arise [17] that can also elicit a stress response. Moreover, individual intrinsic factors such as age, gender, breed, inherited temperament, experience, and previous training [11] are known to impact stress biomarkers.

Cortisol production is the end-result of the hypothalamic-pituitary-adrenal (HPA) axis activation induced by any psychological or physical stressor. It has been studied extensively in horses to quantify stress levels and the response to different types, intensities and durations of exercise in sport and racehorses [10, 18]. The validation of salivary cortisol (SC) [19] allowed its non-invasive assessment in competition settings, including endurance [20–22], showjumping [15, 23, 24] and dressage. Even if a circadian rhythm has been demonstrated, plasma levels did not always correlate with salivary cortisol [25]. The difference could be explained by only the biological active unbound component being present in saliva, whereas, in plasma, both the inactive and active free constituents of cortisol are measured and not necessarily proportional [26]. Cortisol showed greater variations in saliva than in plasma [19, 27]. The highest variations from pre-exercise in salivary cortisol (SC) levels were registered in endurance (up to 1000%) [20] followed by eventing (240%) [28], showjumping (150–340%) and dressage (200%) [29] competitions.

The changes in circulation associated with the HPA axis activation induce periorbital warming that can be quantified by thermal imaging cameras [10]. The use of hairless vascularised areas such as the lacrimal caruncle to measure temperature minimises interference from skin and coat colour, and environmental conditions [30]. The rise in eye temperature measured by infrared thermography (IRT^ET^) was reported as a reliable indicator of short-term stress, and is often studied together with salivary cortisol in horses [10, 14, 31–34]. It has been generally accepted that a rise in eye temperature represents an emotional response to stressors, including exercise [35], as opposed to a physiological response to exercise’s physical demands, as proposed recently [31]. IRT^ET^ may represent a measure of emotive reactivity to effort, that can have a beneficial or detrimental effect on performance [14, 35]. For this reason, IRT^ET^ has recently been proposed as a selection tool to help identify emotional reactivity as a desirable, or undesirable, trait for performance according to the horse’s intended use [35, 36]. The complimentary use of salivary cortisol and IRT^ET^ as non-invasive biomarkers of stress during endurance competitions could help characterise distress and physiological response to effort for endurance horses during exercise in competition.

To our knowledge IRT^ET^ alone or concomitantly with SC has not been studied before during endurance rides. This study aimed to investigate the usefulness of these biomarkers to manage competitions more effectively and monitor horse welfare in endurance competitions. Based on previous studies, it was hypothesised that pre-exercise baseline SC and IRT^ET^ would be higher in younger or less experienced horses, and that post-exercise variation from baseline would be higher in the top finishers.

## Results

### Horse’s previous experience and competition outcome

Age was not significantly different between the 40 km (40K) (median ± IQR = 6.0 ± 1.5) and 80 km (80K) categories (median ± IQR = 6.0 ± 3.0), however, there was a significant (*p* < 0.05) difference in previous experience between the two categories: horses in the 40K had less km in competitions (median ± IQR = 40 ± 30, min = 0, max 120) than horses in the 80K category (median ± IQR = 80 ± 40, min = 80, max = 240). Across all competitions, a total of 11 horses (18%) failed to qualify, six for irregular gait, two for metabolic reasons and the remaining three for other reasons. The speed median (±IQR) was 14.9 km/h (±2.5) and 15.7 km/h (±1.0) for the 40 and 80K categories, respectively. In the first phase, horses in the 40K covered 20 km at a significantly (*p* = 0.006) slower speed (median ± IQR =14.0 km/h ± 1.8), when compared with those in the 80K ride category, that covered either 30 or 40 km (median ± IQR =15.1 km/h ± 0.9).

### Visual assessment of saliva samples

The saliva samples were subjectively judged to have less volume with the progression of the ride. Also, many samples were contaminated with food particles that horses kept in the mouth during the ride phases.

### Age and gender impact in SC and IRT^ET^

No significant differences or correlations were identified between SC or IRT^ET^ with age or gender, except for mares that showed a significantly higher SC (*p* = 0.037) at the final VG3 after 80 km covered (VG3@80km) in the 80 K-B ride.

### SC and IRT^ET^ measurements

**Fig. 1:**
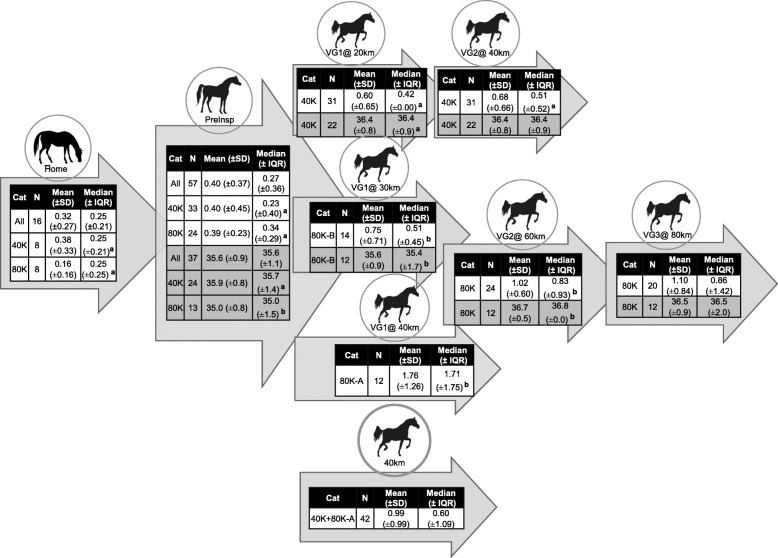
Means and medians of salivary cortisol in ng/dl (white background) and Infrared Thermographic Eye Temperature in Celsius degrees (grey background) of  all horses (ALL), in 40K and 80K categories, collected at Home, Preinspection (PreInsp), by Vet Gate (VG) and covered distance (@). Significant differences (*p* < 0,05) among ride categories at Vet Gates (columns) are signalised by different letters

Means and medians of SC and IRT^ET^ of all individuals collected at the different moments (previous and competition day) are displayed in Fig. 1.

#### Baseline values

The lowest SC levels were registered in all categories at Home or PI. When comparing the 40 K with the 80 K horses’ baseline SC values, there was not a significant difference at Home nor the PI. At the PI, IRT^ET^ was, however, significantly higher (*p* = 0.007) in the horses competing in the 40K (median ± IQR = 35.7 ± 1.4) when compared to those in the 80K (median ± IQR = 35.0 ± 1.5) category ride (Table 1).

**Table 1:** Correlations of Salivary Cortisol (SC) and OT Eye Temperature (ET) measured by Infrared Thermography with performance data in the 40 K and 80 K ride categories

Spearman Rank Correlation				
SPEED	Rho	N	*P*-Value	Ride Cat
**PHASE 1**				
∆SC Home-PI	- 0,7*	10	0,023	40 km
ET PI	- 0,6	13	0,024	80 km
∆ET PI-VG60 km	+ 0,6*	12	0,027	80 km
∆ET VG60- VG80 km	+ 0,6*	12	0,037	80 km
**PHASE 2**				
∆SC PI-VG30 km	+ 0,6	13	0,047	80 km
ET VG3	+ 0,6	12	0,045	80 km
∆ET PI -VG60 km	+ 0,7*	12	0,015	80 km
∆ET VG30 -VG 60 km	+ 0,7*	10	0,033	80 km
**PHASE 3**				
ET VG3	+ 0,6*	12	0,12	80 km
∆ET PI-VG60 km	+ 0,7**	12	0,009	80 km
∆ET PI-VG80 km	+ 0,7*	12	0,033	80 km
**AVERAGE**				
ET PI	- 0,6*	11	0,038	80 km
ET 20 km	- 0,5*	22	0,024	40 km
ET 80 km	+ 0,7*	11	0,024	80 km
∆ET PI-VG60 km	+ 0,8**	11	0,006	80 km
∆ET PI-VG80 km	+ 0,7*	11	0,035	80 km
**RECOVERY TIME**				
**VG1**				
ET 60 km	- 0,6*	12	0,034	80 km
∆ET VG0-VG80 km	+ 0,6*	12	0,036	80 km
**VG3**				
∆SC VG0-VG30 km	- 0,6*	13	0,047	80 km
∆ET VG30-VG80 km	- 0,8**	10	0,009	80 km
∆ET VG60-VG80 km	- 0,6*	12	0,028	80 km
**QUALIFICATION**				
**CL vs FTQ**				
ET PI	−0,5*	24	0,026	40 km
**POSITION**				
SC PI	−0,5*	25**	0,025	40 km
ET VG80 km	−0,7*	12	0,011	80 km
∆ET PI-VG60 km	−0,8**	12	0,005	80 km
∆ET PI-VG80 km	−0,6*	12	0,034	80 km

*p*-value is two-tailed * (*p* < 0,05) **(*p* < 0,001). PI (Pre-Inspection), VG (Vet Gate); 20, 30, 60 and 80 (distance covered in km. ∆ (variation between moments of collection)

#### Analysis by vet gate

The highest SC levels were registered in the first Vet Gate after 30 or 40 km covered (VG1@30/40km) in the 80K ride category, but only in the second or final Vet Gate after twice twenty km covered (VG2@40km) in the 40 K category. In contrast, the highest IRT^ET^ was obtained in VG2 in both ride categories, independently of the covered distance, i.e. 40 or 60 Km. When comparing ride categories at VG1, the 40K horses had a significantly lower SC (*p* = 0.006), but a significantly higher IRT^ET^ (*p* = 0.023), than the 80K horses.

#### Analysis by covered distance

When comparing the same covered distance among ride categories, horses in the 40K having performed two phases of 20 km, with a rest period in-between, showed in VG2 a significantly (*p* = 0.001) lower SC, when compared with those in the 80K ride that had raced uninterruptedly 40 km in one phase and were at VG1.

#### Analysis by final outcome (completion vs failing to qualify: FTQ)

There were no significant differences in SC or IRT^ET^ measurements between the horses that completed the ride and those that failed to qualify, in none of the evaluated moments.

#### Analysis by classification group

**Fig. 2:**
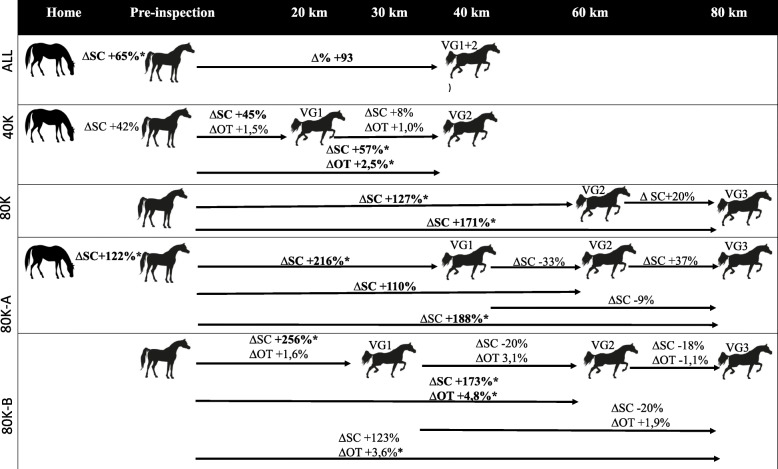
Variations of SC (Salivary Cortisol) and IRTET (Eye Temperature measured by Infrared Thermography) of all horses (ALL), 40 km and 80 km categories, from and in-between Home, Preinspection and covered distances at each Vet Gate. The variations represented between Home and Preinspection represent the sample of horses collected both at home and at the pre-inspection. The variations between Pre-Inspection and covered Km represent the same sample of horses collected at the respective covered distances, thus eliminated horses or horses that failed a collection were not compared. Significant variations among coincident points-in-time and/or covered distance of all horses (ALL), 40 km, 80 km, 80 km-A and 80 km-B categories are in bold signalised by * (*p* < 0,05)

SC or IRT^ET^ levels analysed by classification groups and its evolution across Vet Gates in both ride categories, e.g. horses positioned in the top five (Top5), from 6th to 10th (G2) and from 11th to 15th (G3) can be consulted in Fig. 2. Horses in the 40K competition classifying in the Top5 showed at the PI, significantly higher (*p* = 0.05) SC levels (median ± IQR = 0.90 ng/ml ±0.61) when compared to horses positioned in G3 (median ± IQR = 0.16 ng/ml ±0.40). On the other hand, horses classifying in the Top5 in the 80K competition, demonstrated at VG2, significantly (*p* = 0.05) lower SC levels (median ± IQR = 0.70 ng/ml ±1.00) than horses positioned in G2 (median ± IQR = 1.88 ng/ml ±1.00) and at VG3 (final), a significantly (*p* = 0.053) higher IRT^ET^ (median ± IQR = 37.60 °C ± 0.00), than horses positioned in G3 (median ± IQR = 35.70 °C ± 1.00).

### Variations of SC and IRT^ET^ between collection moments

**Fig. 3:**
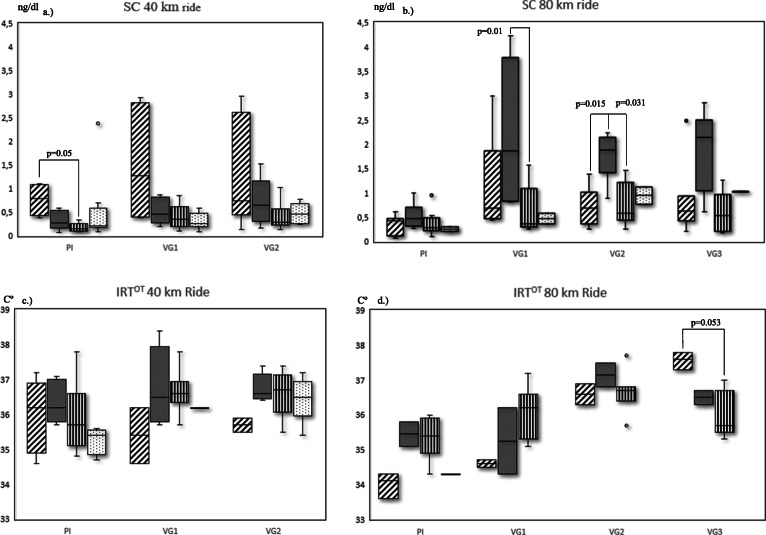
Clustered boxplots of SC (Salivary Cortisol) and IRT^ET^ (Eye Temperature measured by Infrared Thermography) by group position. a.) Salivary Cortisol (SC) in 40 and b.) 80 km rides, and c.) Infra-Red Thermography Eye Temperature (IRT^ET^) in 40 and b.) 80 km rides with horses grouped in Top5 (1th–5th), G2 (6-10th), >11th and FTQ (Failed to Qualify), aligned from left to right, at Preinspection (PI) and Vet Gates (VG).Horizontal X-Axis: Vet Gates (VG); Vertical Y-Axis: SC in ng/dl and IRT^ET^ in centigrade Cº

The magnitude of variations in SC levels and IRT^ET^ between Home, PI and Vet Gates of different ride categories and its significance is shown in Fig. 2. The baseline SC variation between Home and next day PI was only significant (*p* = 0.017) for those horses participating in the 80 K ride category with a 122% rise. The highest SC variation was between PI and VG1, but only significant, in the 80K ride category with a 216 and 256% rise in 80 K-A and 80 K-B, respectively. IRT^ET^ rises were only significant when values were compared across more than one Vet Gate. When analysed by classification group, horses classified in the Top5 (median ± IQR = 33.9 ± 0.0) and in G3 (median ± IQR = 35.3 ± 1.0) had a variation of 10.65 and 1.78% from the PI to VG3, respectively (Fig 3).

### Correlations between SC, IRT^ET^ and performance

Significant correlations between SC or IRT^ET^, its variations and performance data (speed, recovery time, final position) are presented in Table 1. An association could not be established between SC baseline values, nor its variations from Home to PI, with the outcome. However, when analysed by classification group, a classification in the Top5 of the 40K  ride category was significantly (*p* < 0.05) associated with an IRT^ET^ decrease from PI to VG2. In contrast, in the 80K ride category, a lower IRT^ET^ at the PI was significantly (p < 0.05) associated with a faster speed in phase 1, overall average speed and completion. Also, the higher the IRT^ET^ variation from PI into VG2 and VG3 was associated with a better placement.

Correlations between SC and IRT^ET^ were scarce and are depicted in Table 2.

**Table 2:** Significant correlations found between SC (Salivary Cortisol) and Eye Temperature (ET) measured by Infrared Thermography in the 80 km category

Spearman Rank Correlation			
	Rho	N	*P*-Value
**SC 40 km**			
ET 60 km	- 0,9*	5	0,037
**∆ SC VG30-VG60 km**			
∆ET PI-VG30 km	- 0,8*	8	0,032
∆ETET PI -VG80 km	- 0,8*	9	0,012
**∆SC VG40-VG80 km**			
∆ET VG60-VG80 km	+ 0,8*	6	0,050
**∆SC VG60-VG80 km**			
∆ET VG60-VG80 km	- 0,8*	6	0,050

*p*-value is two-tailed * (*p* < 0,05) **(*p* < 0,001) PI-Pre-Inspection), VG (Vet Gate); 20, 30, 60 and 80 km (distance covered in km. ∆ (variation between moments of collection)

## Discussion

Endurance riding evolved in the last two decades from an amateur activity into a highly professionalised sport. Better training techniques and more specialised breeding allowed the creation of equine endurance super-athletes, capable of achieving a sustained high speed along with a fast-cardiac recovery capacity. This preliminary study aimed to determine how salivary cortisol (SC) and eye temperature measured by infrared thermography (IRT^ET^) and their variations before and during endurance competitions were related to the outcome and performance of competing horses, and their potential usefulness in depicting compromised horses.

### Behaviour of SC and IRT^ET^ during competitions

Various factors inherent to competitions, such as accustoming to a novel environment [37] and a new group of horses [38] or undergoing a veterinary examination [39] have been described as potential stressors to horses. Transportation is considered a major stressor capable of generating greater SC rises than exercise [13]. Even in short distances such as 1 h, a 4-fold SC increase was previously reported [12]. All horses in our study were transported to the venue the same morning of the competition, arriving typically near the time of the PI and the estimated transportation time ranged between 10 min and no longer than 2 h. In this study, an overall 65% SC increase from the baseline values the eve at Home to the first collection performed at the competition venue immediately after the PI, was modest and less than the competition itself’s rise.

Higher cortisol rest levels [13, 24] and IRT^ET^ [33, 35] were previously reported in younger or less experienced horses. However, basal cortisol levels have also been reported to be similar in a competition setting between horses with different experience levels [40]. Even if there was not a significant difference in SC levels at Home or the PI among ride categories, in our case, IRT^ET^ was higher in the less experienced horses participating in the 40 K ride in the PI. Both SC and IRT^ET^ have been used as indicators of distress in non-exercised horses [34]. Eye temperature is considered a more immediate stress indicator than cortisol, reported to take at least 15 min to increase after exposure to a stressor [10]. Since IRT^ET^ was measured immediately after exiting the VG, this could reflect a higher distress of the 40 K horses exposed to the veterinary examination and, often, being separated from their mates at the PI.

As expected, both SC and IRT^ET^ lowest values were registered at Home and at the PI. However, regarding the occurrence of the highest values there was a difference between ride categories. In the 40K ride the maximum SC and IRT^ET^ were registered in the final Vet Gate, as opposed to the 80K rides, where they were obtained at mid-distance in VG1 and VG2, respectively but not in the final Vet Gate.

Our study corroborates that both intensity and duration, if uninterrupted, contribute to SC increase [41]. Indeed, those horses in the 80 K category that performed a straight 40 km phase into VG1 at a higher speed showed a 3-fold higher cortisol level than horses in the 40K  ride, which raced two 20 km phases with a rest period in-between.

Our study agrees with previous studies performed during endurance competitions, which also registered the highest SC increases in the first half of the rides [20–22]. Regardless of the covered distance and registered levels, the steepest SC variations took place in both ride categories in VG1, showing much more modest, or even negative variations, in the subsequent VGs. This effect was also reported previously in human athletes, whose cortisol levels increased after short-term and decreased after prolonged, i.e. lasting several hours, exercise [42]. This drop is believed to result from the negative feedback system generated by the high cortisol levels induced by exercise. Two mechanisms were proposed for athletes to bypass the negative feedback. First, interleukin-6 released from working muscles induced by low glycogen contents seems to act as a hormone, stimulating, similarly to cortisol, the maintenance of glucose homeostasis during exercise and mediating exercise-induced lipolysis [43]. The second mechanism could be the individual’s inherent ability to override the serotonergic mechanisms (that inhibit CRH release and therefore the HPA axis) involved in central fatigue, which is not necessarily related with training level [44]. This drop could also be connected to a decrease in the first moment from a decrease in horses’ emotional stress content. It was also proposed before that the initial higher levels could be associated with excitement and not with body demand [20]. The emotional stress could, therefore, explain the variations reported in other studies at similar magnitudes, but in much lighter exercises [29].

IRT^ET^ was used to characterise stress levels induced by certain equestrian practices such as neck hyperflexion [14] or a tight noseband [45]. More recently, IRT^ET^ was also studied in showjumping [33, 46] and dressage competitions [36], in Standardbred harness races [35] and in flat race Arabian and Thoroughbred horses in training [32]. One of the proposed added values of the use of IRT^ET^ is its potential independence from the effort effect, thereby providing a valid means of evaluating the emotive reaction to effort stressors in exercised horses [10].

We could only find very few associations between SC and IRT^ET^. This is in line with other studies that investigated SC and IRT^ET^ simultaneously during exercise [14, 32, 33, 35, 47]. One study could establish an association between the two biomarkers in exercise, but only after an ACTH stimulating challenge test [48] and another, during clipping, a non-exercise activity [34].

In our study, the highest IRT^ET^ rise in consecutive Vet Gates was from VG1 to VG2 (+ 3.1%) in the 80 K ride, that also corresponded to the highest SC drop (− 20%). This might be explained by cortisol representing mainly the physiological response to exercise, and the eye temperature, the prolonged effort’s emotional reactivity.

### SC levels and IRT^ET^ association with competition outcome

Elevations of basal cortisol concentrations in response to emotional stress are believed to be detrimental to general health, but not necessarily to sport performance [49]. Indeed, in the more inexperienced horses of the 40K ride, the higher SC levels before and during the ride were associated with better performance, reflecting most likely the extra necessary physiological response to effort (Table 1 and Fig. 1). In the 80 K category, cortisol behaved differently. It appears it was not the pre-exercise SC level that influenced the results per se, but the magnitude of increase from PI to VG1@30km associated with a higher placement group (Table 1). Moreover, the group finishing in the Top5 showed a significantly lower SC than the slower G2 in the second-to-last vet gate or VG2. This may indicate an extra effort in less well-prepared horses of G2. Cortisol was shown to increase with effort intensity, but in horses subjected to the same amount of exercise, the rise was higher in untrained horses [50].

IRT^ET^ was proposed as an alternative biomarker capable of quantifying emotional reactivity to effort, instead of a direct measure of effort like cortisol [35, 36, 46]. A lower and higher IRT^ET^ before and after exercise, respectively, i.e., a higher variation after exercise, was reported to be associated with better performances by analysing 130 Spanish Standardbred horses in harness races [35]. The same authors concluded that a variation of − 0.97% represented the break-point under which physiological stress developed. In our study, the 80K category horses with a lower IRT^ET^ at the PI and a more significant rise into the final VG3 were better placed in the final classification (Table 1). Furthermore, this rise was associated with a shorter recovery time in VG3, but not in VG1, which might be attributed to the initial excitement. The 40K ride horses showed very few associations with IRT^ET^. A reason for that could be that they started with an already higher IRT^ET^ at the PI. Negro et al. [35] estimated a pre-race eye temperature of 37.61 ± 2.85 C °C with a post-race variation of + 7.57% as the optimal values for performance. Our lower number of horses and the prolonged low-intensity non-explosive nature of endurance exercise, precluded these calculations. Yet, horses classified in the Top5 when compared with G3 had an average IRT^ET^ of 33.8 °C and 35.33 °C with a variation of + 10.65% and + 1.78%, respectively.

More studies are warranted to investigate the meaning and usefulness of IRT^ET^.^.^A recent study proposed IRT^ET^ as an indicator of physical fitness in ranch horses [31], as opposed to a purely psychological reaction to effort. The rise was attributed to increased blood flow in muscles and peripheral heat dissipation. A correlation was found with creatine kinase (CK), indicating a possible association with muscle damage.

### Failure to qualify

In this research, most likely due to the small sample, we could not find a difference or association between eliminated or classified horses and SC levels or IRT^ET^.

#### Limitations of the study

##### Volume and food contamination in SC determination

In this study, we used the saliva collection protocol described by Peeters et al. (2001). Therefore, in further endurance studies, we recommend that due to the horses’ progressive natural dehydration, which likely justified the diminished saliva volume observed as the competitions progressed, an increase of the Salivette’s® contact time with the oral cavity along with the progression of a ride. How the level of salivary free cortisol is affected by reduced saliva warrants investigation [25]. High and low flow rates in normal adult humans did not show a difference in concentration in SC [51]. Even though the sample was smaller in this study, the highest increases of SC concentration still occurred in VG1, when horses were supposedly less dehydrated, and not in VG3.

It was also noticed that many saliva samples after extraction were contaminated with food. To investigate possible interference with the results a small trial was performed in five horses after a mouth wash to compare clean saliva and saliva posteriorly contaminated and incubated with different types of food (hay, granulated and grass). No significant differences (*p* < 0.05) were found between the different samples (MM et al. 2019, unpublished data). A recent study also showed that food contamination did not alter SC levels significantly [52].

##### Non-controlled interferences with IRT^ET^

The same operator recorded IRT^ET^ measurements during the research study and distance from the operator to the eye was measured at all times. However, we recognise that our values might have been affected by the environmental conditions’ interference throughout the day. Ambient conditions, surface moisture, brightness, sun reflection and wind breeze are some of the variables that have been reported to interfere with IRT shooting [10]. A controlled environment as recommended [53] is challenging to achieve in endurance competitions, without interfering with the competition’s pace and time management of the competitors.

#### Other parameters not quantified

Even if disrupted, how circadian rhythm could have influenced the variations from PI into VG1 was not taken into account. Also, the impact of different transportation times and characteristics, even over short-distances, could impact basal SC and IRT^ET^ was not quantified during travelling. Trainers were not questioned about the previous training of their horses. Prior competition history, including completions/eliminations rates and previous speed/recovery times/position records, were not analysed.

Measurement of body temperature was not included in this study due to its perceived invasiveness and practicality in young horses and less experienced horses participating in qualifier rides. Soroko et al. (2016) did not find a post-exercise correlation between rectal temperature and maximum eye temperature in 19 racehorses.

The rider’s riding skills and weight might affect the emotional or physical response, e.g., the horse’s capability to cope more or less efficiently with the demanded effort. In one study, SC was not affected by the rider’s experience in showjumping [54], but the weight was found to have a detrimental effect on equine gait and behaviour. Furthermore, many times in endurance, the competitor might be just the pilot for that competition and not the rider training the horse, and whether this causes more stress to the horse was not studied before.

#### Future directions

It is still challenging to untangle emotional distress and experienced pain from the natural physiologic response to the effort. To exhaust the topic usefulness of biomarkers in identifying horses at risk during endurance competitions, more extensive studies are needed at high-level competitions, to collect statistically significant samples of horses that failed to qualify.

## Conclusion

Pre-exercise baseline IRT^ET^ levels, but not SC, were higher in less experienced horses in the 40K compared to their counterparts in the 80K competitions. SC and IRT^ET^ showed different indications according to competition. In the 40 K competition, higher baseline pre-exercise SC levels seemed to be linked to a better classification outcome. In contrast, in the 80 K horses, the higher IRTET variation from pre-exercise into final Vet Gate was the parameter associated with a better performance. A more controlled environment and a larger sample are needed to confirm these results and monitor horse welfare in competitions.

## Methods

### Horses

After competitors, owners and trainers were notified of the aims and methods of the research study to ensure informed consent, a convenience sample of 61 out of 110 horses participating in two endurance events in Portugal was obtained. Age was between 6 and 11 years and 24 were geldings, 29 were mares and eight were entire males. Breed varied; 30 horses were registered as Arabian, 27 as Anglo-Arabian or Part-Arabian and four as other breeds (one Lusitano and three from undetermined origin) in the Portuguese National Federation on-line database (www.fep.pt). All horses were transported the same day to the competition sites and travelling times were estimated according to their training stables’ location.

### Sampling moments and competition’s features

Horses were sampled at home and at two competition sites at different times of the same year, e.g. June and November. At the Polo da Mitra of the University of Evora (MI) competition site, the saliva for cortisol determination of 23 horses was sampled. Of those, 14 horses were collected at their stables (Home), 22 to 24 h before the start of the event, depending on the owner or trainer’s availability. At Torre de Palma Resort in Monforte (TP), 38 horses were sampled for cortisol and eye temperature was measured. Of the 61 horses that entered the study, 34 and 25 horses were participating in 40, and 80 km controlled speed (up to 16 km/h) qualifier rides, respectively, and only two horses were in an 80 Km free speed competition. For data processing competitors were grouped under 40 and 80K categories only.

The sampling took place following the veterinary inspections at the pre-inspection (PI) and upon completion of each phase immediately after the horses exited the Vet Gate area (VG), which was outdoor. If the horse failed to meet the heart rate criteria of 64 bpm, the collections were made after the heart rate reinspection. Requested or compulsory reinspections data were not used. The PI commenced at both sites at 7:00 AM and starts into the track took place in a staggered manner from 8:00 AM for the 80 km and 9:00 AM for the 40 km rides. The competitions finished around 3:00 PM.

Both 40 km qualifier rides were composed of two phases of 20 km, being the cumulative distance at VG1 20 km (VG1@20 km) and at VG2 40 km (VG2@40km). The 80 km rides had three phases, but with a different configuration between competition sites. At MI (80 K-A), the first phase had 40 km (VG1@40 km) and the remaining two had 20 km (VG2@60km and VG3@80km). At TP (80 K-B), there were two phases of 30 km (VG1@30km and VG2@60km) and only the last phase of 20 km (VG3@80km). Of the 61 horses that entered the study, 34 were competing in 40 km and 27 in 80 km rides.

### Collection of saliva

A Salivette® (Starsted) synthetic swab was held on a metal clamp and maintained in every participating horse’s mouth for 30–40 s, over and under the tongue, as described [28], and then placed into the Salivette® (Starsted) tube to be stored at 4 °C, at each collection moment. At the end of each day, the Salivettes were centrifuged for 10 min at 1500 *g* for saliva extraction and stored at − 28 °C until assayed. After thawing the samples, free cortisol was determined using a double-antibody immunoassay kit (Cortisol ELISA, IBL International GMBH, Germany).

### Infrared thermographic eye temperature (IRT^ET^)

Eye temperature was measured using a portable infrared thermography camera (Thermal Imaging Camera, E60BX, FLIR Systems AB, Sweden) with 320 × 240 pixels set to emissivity 0.98. The sampling was performed outdoors in an open field after the horses exited the Vet Gate and before the saliva collection. To calibrate the camera results, environmental air temperature, and relative humidity were measured with a digital thermohygrometer (MR77, FLIR systems AB) at each collection. The left eye was scanned at a 90° angle at a distance of 1 m, as described previously [33], and several images were obtained. After electing the most adequate picture, an image analysis software (ThermaCam Researcher Pro, FLIR systems AB) was used to measure the maximal temperature within an oval area traced around the inner canthus of the eye, including the lacrimal caruncle at ~ 1 cm around the outside of the eyelids [55].

### Performance and outcome data

Outcome and performance data (speed, recovery time and classification) were obtained from the veterinary cards and timing system. For analysis purposes, groups were created according to final position: Top5 (1 to 5th), G2 (from 6th to 10th^)^ and G3 (from 11th). Those that failed to qualify were grouped under FTQ.

### Data analysis

SPSS® version 22 software (Armonk, NY: IBM Corp.) was used for descriptive analysis and inferential statistics.

Variations in SC (∆SC) were calculated as the percent of variation from one moment of collection to the following, according to the following formula:

$$ \Delta  \mathrm{SC}=\frac{Mean\  SC\ \left(t+1\right)- Mean\  SC(t)}{Mean\  SC(t)}\ x\ 100 $$

Where t is a determined moment of collection and t + 1 the following moment of collection. Variations in IRT^ET^ (∆IRT^ET^) were calculated in the same manner. Since data did not assume a normal distribution, a series of Kruskal Wallis analyses with post hoc Mann Whitney U tests identified significant differences between the variables recorded across ride categories, site, breed, and gender. A Wilcoxon signed-rank test assessed if cortisol and IRT^ET^ or variation of each measure were significantly different between collection moments. Where significance was found, post-hoc Bonferroni t-tests were used for multiple pairwise comparisons. A series of Spearman Rank Order correlations analysed if cortisol and IRT^ET^ were impacted by age and gender, speed and classification of the horses. Analysis significance was set at *P* < 0.05.

## Data Availability

The datasets are available from the corresponding author on reasonable request.

## Abbreviations

40K: 40 km endurance ride
80 K: 80 km endurance ride
Δ: Variation
FEI: Fédération Equestre Internationale
FTQ: Fail to Qualify
G2: Horses that were placed from 6th to 10th position
G3: Horses that were placed from 11th to 15th position
HPA: Hypothalamic-pituitary-adrenal
IRT^ET^: Eye temperature measured by infrared thermography
MI: Polo da Mitra of the University of Evora competition site
PI: Pre-Inspection
ROC: receiver operating characteristic curve analysis
SC: Salivary cortisol
Top5: Horses that were placed in the first five positions
TP: Torre de Palma Resort competition site
VG: Vet Gate
VG1@20km: First vet gate after 20 km in competition
VG1@30km: First vet gate after 30 km in competition
VG1@40km: First vet gate after 40 km in competition
VG2@40km: Second vet gate after 40 km in competition
VG2@60km: Second vet gate after a 60 km in competition
VG3@80km: Third vet gate after a 80 km in competition
